# Simultaneous dermatophytosis and keratomycosis caused by *Trichophyton interdigitale* infection: a case report and literature review

**DOI:** 10.1186/s12879-019-4612-0

**Published:** 2019-11-21

**Authors:** Mingrui Zhang, Lanxiang Jiang, Fuqiu Li, Yangchun Xu, Sha Lv, Bing Wang

**Affiliations:** Department of Dermatology, the Second Hospital of Jilin University, No. 218, Ziqiang street, Nanguan district, Changchun, 130000 China

**Keywords:** Dermatophytosis, Keratomycosis, *Trichophyton interdigitale*, Case report

## Abstract

**Background:**

Dermatophytosis is a fungal infectious disease caused by dermatophytes, which produce protease and keratinase to digest keratin, leading to the colonization, invasion, and infection of the stratum corneum of the skin, hair shafts, and nails. *Trichophyton interdigitale* belongs to *Trichophyton mentagrophytes* complex, which is the common pathogen causing dermatophytosis. Fungal keratitis, also called keratomycosis, is an infectious disease of cornea.

**Case presentation:**

Here, we report a case of simultaneous dermatophytosis and keratomycosis caused by *Trichophyton interdigitale*. A 67-year-old man presented with extensive erythema all over the body since 4 years ago, fungal infection of left eye for 2 years, and loss of vision in the eye. These symptoms had become aggravated in the last month. Dermatological examinations showed extensive erythematous plaques with clear borders and scales, scattered red papules with ulceration, and scabs throughout the body. Onychomycosis was observed on the nails of left hand, conjunctival infection with secretion and loss of vision were noted in left eye. Hyaline septate hyphae were observed under direct microscopic examination, fungal culture and internal transcribed spacer sequencing revealed *T. interdigitale*. Histopathological examination suggested infectious granuloma. A diagnosis of dermatophytosis and keratomycosis caused by *T. interdigitale* with loss of vision in left eye was made. The patient was treated with luliconazole cream (two applications per day) and itraconazole (100 mg, *BID*, *PO*). Complete clinical remission was achieved after 1 month. Subsequently, the patient underwent left eye enucleation in the ophthalmology department.

**Conclusions:**

In the present study, we reported a case of simultaneous dermatophytosis and keratomycosis caused by *T. interdigitale*, and reviewed the literature on corneal infection caused by *Trichophyton.* A total of 10 articles with 45 patients were published between 1973 and 2018. The pathogen of 27 patient were identified to species level. There were *T. schoenleinii* (17), *T. mentagrophytes* (4), *T. verrucosum* (3), *T. rubrum* (1), *T. erinacei* (1), and *T. interdigitale* (1). Five patients had corneal trauma, one had contact lens use history. Direct microscopic examination, fungal culture, and analysis of physiological characteristics were the main methods of identification. Early diagnosis and prompt treatment may help improve the management and outcomes.

## Background

Dermatophytosis is a fungal infectious disease caused by dermatophytes, which produce protease and keratinase to digest keratin, leading to the colonization, invasion, and infection of the stratum corneum of the skin, hair shafts, and nails [1]. Tinea corporis is a common dermatophytosis that involves smooth skin, except for the scalp, hair, palms, nails, and genital area. The risk factors for extensive dermatophytosis include genetic defects [2, 3], chronic diseases [4, 5], immunosuppressive therapy [5, 6], and misdiagnosis or delayed diagnosis [7, 8]. *Trichophyton interdigitale* is a strictly anthropophilic species that belongs to the *Trichophyton mentagrophytes* complex, which is the common pathogen causing dermatophytosis [9]..

Fungal keratitis, also called keratomycosis, is an infectious disease of the cornea. Low awareness and delayed diagnosis of this condition lead to complications that can result in permanent loss of vision, and even necessitate enucleation [10, 11].

In the present study, we report a case of simultaneous dermatophytosis and keratomycosis caused by *T. interdigitale*, which ultimately led to the permanent loss of vision in one eye.

## Case presentation

**Fig. 1:**
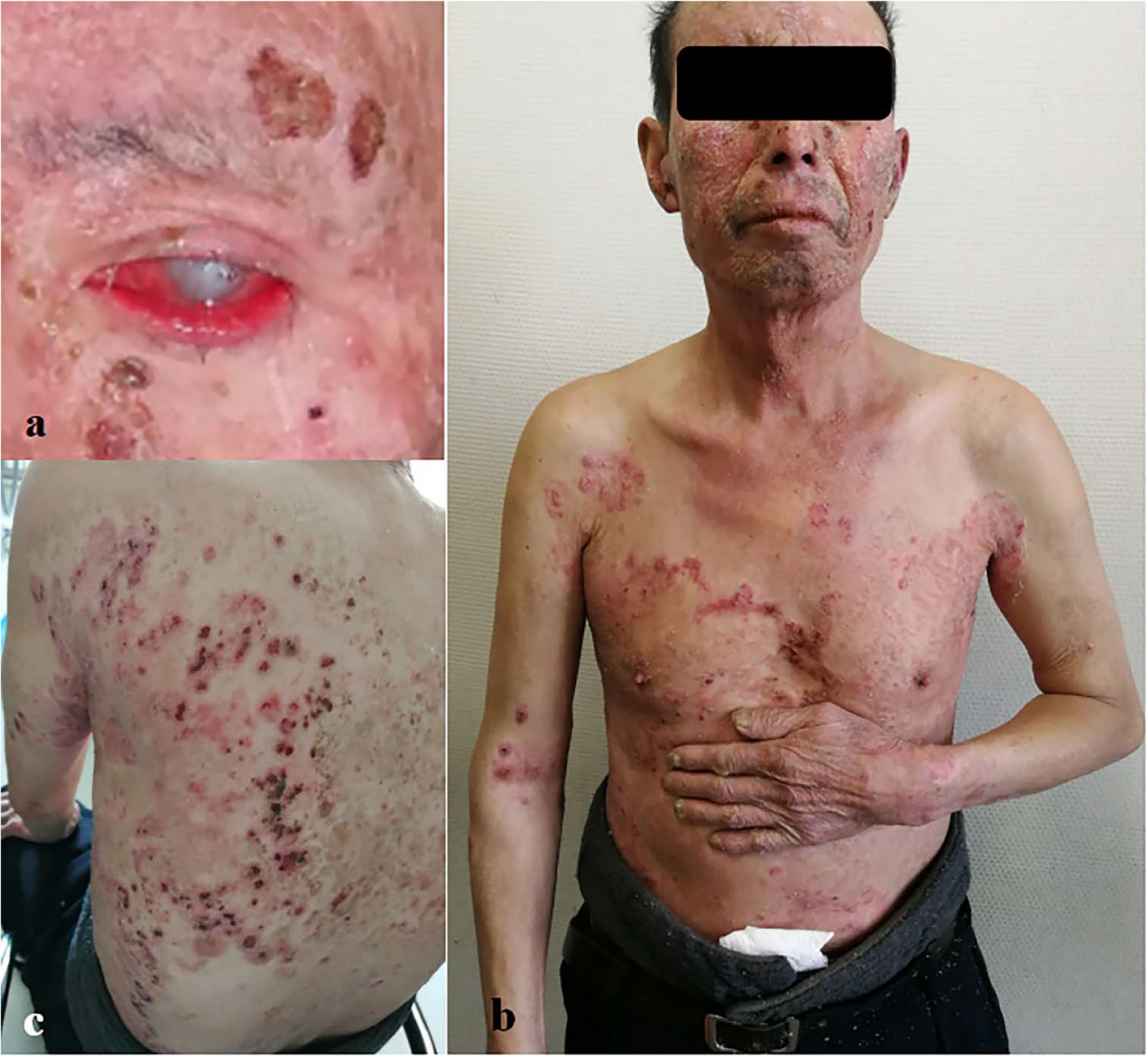
**a** Conjunctival infection with secretion, corneal ulcer, and loss of vision in the left eye. **b** and **c** Extensive erythematous plaques with clear borders and scales, scattered red papules with ulceration, and scabbing throughout the body; onychomycosis can be observed on the nails of the patient’s left hand

A 67-year-old man was admitted to the dermatology department of our hospital with multiple ringworm lesions on his face, trunk, and limbs. The lesions first appeared within inches of his left eyebrow 4 years ago, and then gradually extended across face, trunk and limbs. Two years ago, he was diagnosed with fungal keratitis at a local hospital. About 1 year ago, he lost the vision in his left eye. Cutaneous symptoms had become aggravated in the last month. Dermatological examinations showed extensive erythematous plaques with clear borders and scales, scattered red papules with ulceration, and scabs throughout the body. Onychomycosis was observed on the nails of his left hand. An ophthalmological examination showed conjunctival infection with secretion, corneal ulcer, and loss of vision in the left eye (Fig. 1). The patient complained of mild itchiness over the lesions and pain in left eye. He had no history of diabetes, eye trauma, or any other significant medical disorders. A history of high-risk behaviors (e.g., multiple sex partners and intravenous drug abuse) for acquired immunodeficiency was not present. He had been diagnosed with fungal keratitis complicated by iridocyclitis in other hospitals, and had received irregular antifungal treatments, such as itraconazole and terbinafine. He had a history of multidrug treatment, including corticosteroid, because of the misdiagnosed with psoriasis and eczema.

**Fig. 2:**
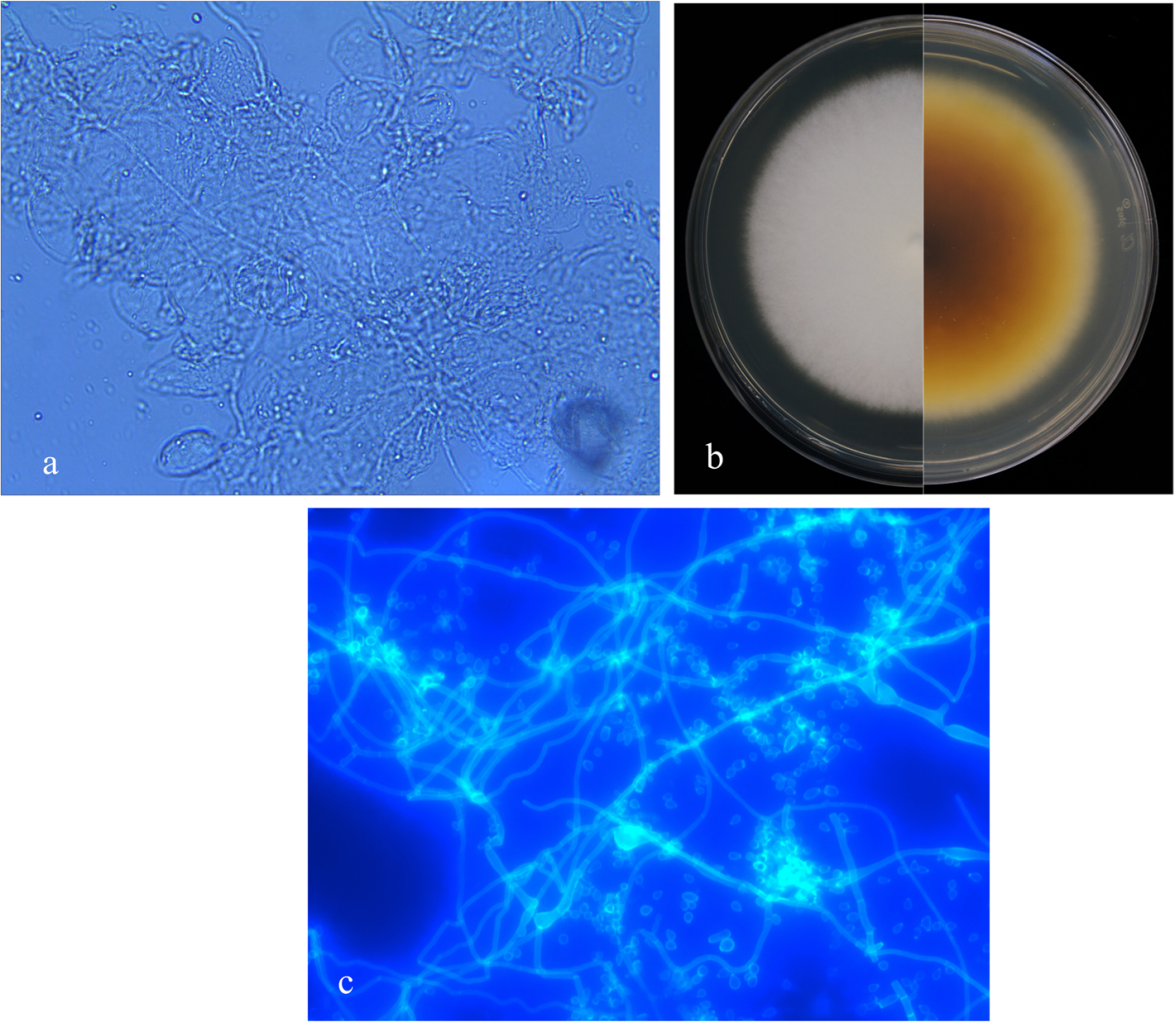
**a** Direct microscopy showing hyaline septate hyphae. **b** Potato dextrose agar plates incubated at 28 °C showing medium growth rate and colonies with white powdery surfaces. The bottom of the colonies turned yellowish-brown, while the surface became granular over time. **c** Slide cultures with calcofluor white stain (× 40) reveal branched septate hyphae and masses of spherical-to-pyriform microconidia

**Fig. 3:**
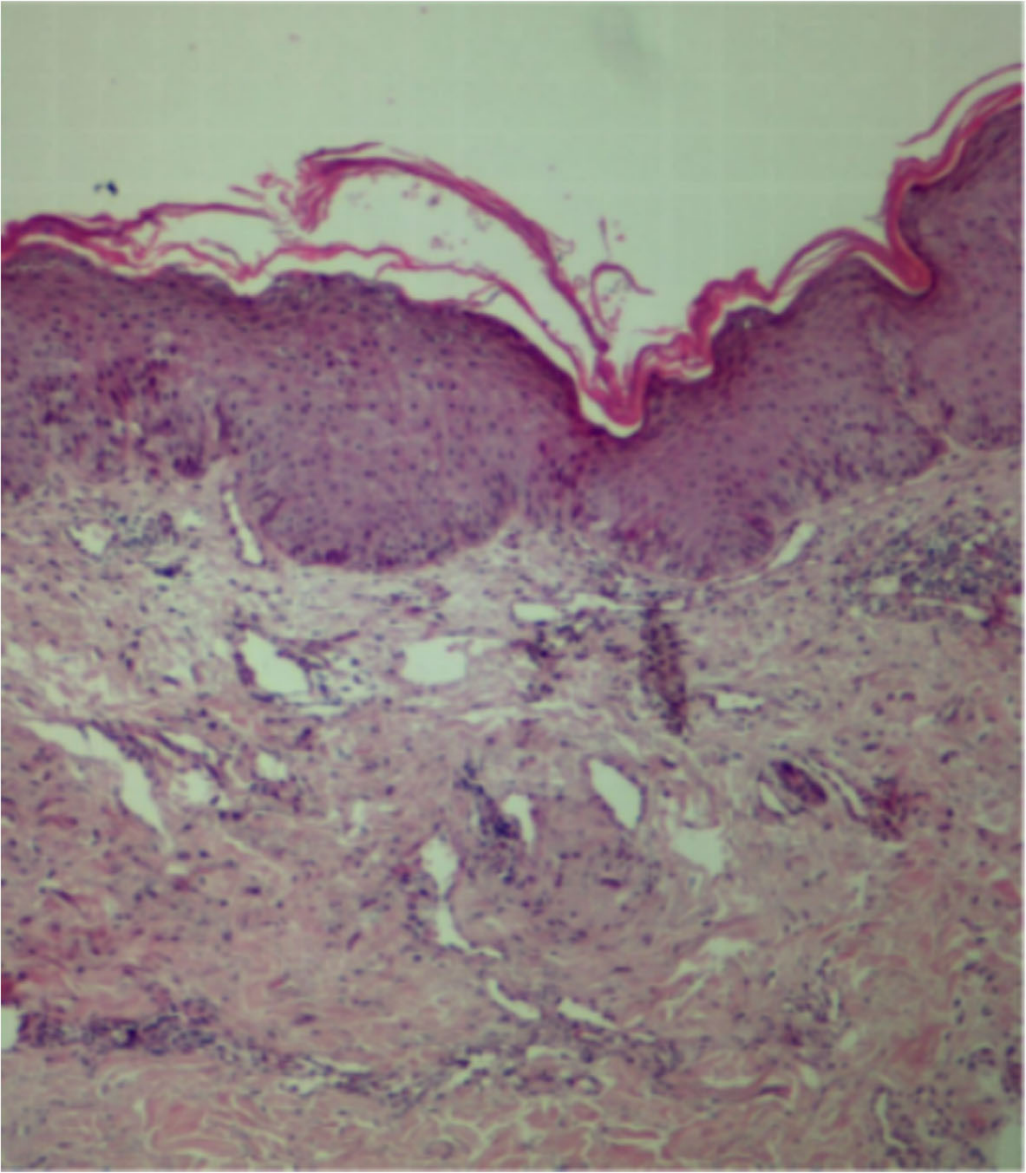
Histopathological examination of a biopsy specimen reveals parakeratosis, mild acanthosis, dense dermal blood vessels, and lymphocyte and plasma cell infiltration (hematoxylin and eosin; × 100)

Direct microscopy with 10% potassium hydroxide revealed hyaline septate hyphae (Fig. 2a). Biopsy specimens from the skin lesions, nail and corneal scrapings were inoculated on Sabouraud dextrose agar containing chloromycetin at 28 °C, and white downy colonies grew. These isolates were then subcultured on potato dextrose agar plates, which showed a medium growth rate and produced colonies with white powdery surfaces (Fig. 2b). Slide culture revealed branched septate hyphae and masses of spherical-to-pyriform microconidia (Fig. 2c). For accurate identification, we extracted genomic DNA of the strains and performed polymerase chain reaction (PCR) assays targeting the internal transcribed spacer region (ITS) using primers and amplification conditions described previously [2]. PCR products were sequenced and compared in CBS (www.cbs.knaw.nl) and GenBank database (https://blast.ncbi.nlm.nih.gov/). The ITS sequence of isolates shared 99% identity with the reference sequence for *T. interdigitale* (CBS 428.63^T^). The minimum inhibitory concentrations (MICs) of eight antifungal agents (SigmaAldrich, St Louis, MO, USA) were determined using Clinical and Laboratory Standards Institute methodology [12]. *T. mentagrophytes* (ATCC MYA 4439), *Candida parapsilosis* (ATCC 22019), and *Candida krusei* (ATCC 6258) were used as quality controls. The MICs of the antifungal drugs terbinafine, micafungin, caspofungin, posaconazole, voriconazole, itraconazole, fluconazole, and amphotericin B were ≤ 0.03 μg/mL, ≤0.03 μg/mL, 0.25 μg/mL, ≤0.03 μg/mL, 0.06 μg/mL, 0.06 μg/mL, 4 μg/mL, and 1 μg/mL, respectively. QC results were under normal ranges. Histopathological examination of biopsy specimen revealed parakeratosis, mild acanthosis, dense dermal blood vessels, and lymphocyte and plasma cell infiltration (Fig. 3). Laboratory tests revealed decreased levels of IgG (4.07 g/L; normal range: 7.51–15.60 g/L), IgA (0.59 g/L; normal range: 0.82–4.53 g/L), CD3 + CD4+ T-cells (15.6%; normal range: 27.0–57.0%), CD4+/CD8+ T-cells (0.20%; normal range: 1.06–2.66%), increased levels of CD3+ T-cells (96.3%; normal range: 61.0–77.0%) and CD3 + CD8+ T-cells (78.5%; normal range: 14.0–34.0%). The other test results were all within normal ranges or negative. Because of the severe clinical manifestations, the abnormal T-cell subsets and immunoglobulin levels, we suspected a genetic defect in the immune response to fungal infections. After fully explaining his options to the patient and his family, written informed consent from the patient for molecular genetic studies was obtained, according to the rules of the Clinical Research Ethics Committee of the Second Hospital of Jilin University. Genomic DNA was extracted from the peripheral blood of the patient. We analyzed all the exons encoding caspase recruitment domain-containing protein 9 (CARD9) [13] and signal transducer and activator of transcription 3 (STAT3) [14], which have previously been linked to invasive fungal infections. And no disease-causing mutation was found in these two genes.

### Diagnosis and treatment

**Fig. 4:**
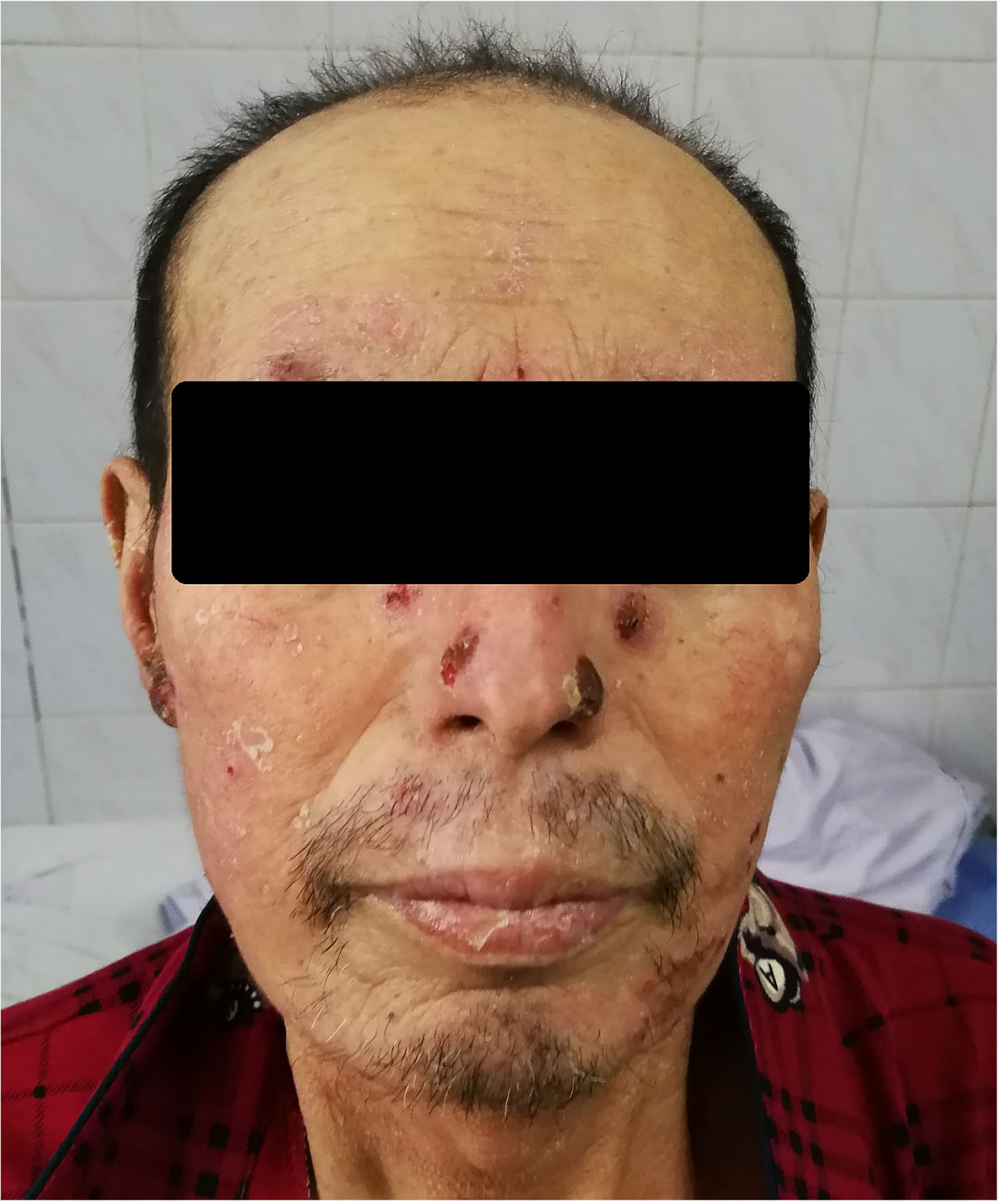
Significant improvement was observed after 14 days

Considering the clinical manifestations and examinations, a diagnosis of dermatophytosis and keratomycosis caused by *T. interdigitale* with loss of vision in the left eye were made. The patient was treated with luliconazole cream (two applications per day) and itraconazole (100 mg *BID*, *PO*) for 1 month. A significant improvement was observed after 14 days (Fig. 4). Subsequently, the patient presented to the ophthalmology department for left eye enucleation. There has been no recurrence during 3 months of follow-up.

## Discussion and conclusions

Tinea corporis is a common type of dermatophytosis that infects smooth skin, except for the scalp, hair, palms, nails, and genital area. When the pathogens invade the stratum corneum of the skin, they cause a mild inflammatory reaction, consisting of erythema, papules, and blisters, followed by ringworm lesions with obvious scales. The source of the infection is typically contact with contaminated items, infected animals [15–18] or spread from an adjacent skin lesion. The risk factors for extensive dermatophytosis involving large parts of the body include genetic defects [2, 3], chronic diseases, such as diabetes, chronic hepatitis, kidney disease, and malignant tumors [4, 5], immunosuppressive therapy, long-term use of corticosteroid [5, 6], and misdiagnosis [7, 8]. The diagnosis of dermatophytosis is mainly based on clinical manifestations and direct microscopic examination. The pathogens can be identified based on culture morphology, physiological characteristics, and molecular sequencing.

*T. interdigitale* belongs to the division Ascomycota, order Onygenales, family Arthrodermataceae, and genus *Trichophyton*. It is a strictly anthropophilic species of the *T. mentagrophytes* complex [9]. Along with *T. rubrum*, *T. interdigitale* is a main causative agent of dermatophytosis, and can even cause dermatophytous granuloma [19] and eye infections [20].

Keratomycosis is a vision-threatening corneal fungal infectious disease that occurs all over the world, and is associated with progressive keratolysis, perforation, scleral extension, and endophthalmitis [21]. Poor visual outcomes are correlated to delays in clinical diagnosis, the virulence of fungal organisms, and limitations in effective antifungal agents [10, 11]. Corneal trauma, the injudicious/unreasonable use of corticosteroids or immunosuppressants, immunodeficiency diseases such as diabetes and AIDS, and the overuse of contact lenses are the major risk factors for the development of fungal keratitis [22]. At present, up to 56 genera and 105 pathogenic fungi that can cause fungal keratitis have been identified [23]. *Aspergillus*, *Fusarium*, and *Candida* species remain the most common organisms isolated worldwide [24]. In China, *Fusarium* is the main pathogen of fungal keratitis, followed by *Aspergillus*, *Penicillium*, and *Curvularia*. Keratomycosis caused by dermatophytes is very rare [20, 25, 26]. Nevertheless, *Trichophyton* spp. are an important entity implicated in fungal keratitis. Case reports from around the world have designated it as a dangerous pathogen [26]. Correct identification, definite diagnosis, prompt and appropriate clinical management play important roles in improving the prognosis of patients with fungal keratitis [27].

In this paper, we reviewed the literatures on fungal keratitis caused by *Trichophyton* spp. to help clinicians and researchers recognize that this genus is capable of infecting the eyes, and is a potent etiological agent of fungal keratitis. All published case reports and retrospective analyses on *Trichophyton*-related fungal keratitis were identified through an extensive search of the PubMed, MEDLINE, CNKI, and Wanfang databases by using different sets of key words, viz. *Trichophyton*, fungal keratitis, and keratomycosis, in both the English and Chinese. After removing duplicate reports, we had a total of 10 articles with 45 patients, published between 1973 and 2018 (Table 1). In 18 patients, pathogen identification was performed down to the genus level, while in the remaining 27 patients, it was performed down to the species level. Among these 27 patients, 17 patients had infections caused by *T. schoenleinii*, 4 patients caused by *T. mentagrophytes*, 3 patients caused by *T. verrucosum*, and 1 patient each had infections caused by *T. rubrum*, *T. erinacei*, and *T. interdigitale*. Five patients had a clear history of corneal trauma, and one patient had a long history of contact lens use. Direct microscopic examination, fungal culture, and analysis of physiological characteristics were the main methods of identification. In 2001, Tang et al. reported a case of keratitis with left eyelids infection caused by *T. mentagrophytes* [20], And in 2014, Jin KW et al. reported a case of keratomycosis with onychomycosis [32]. In addition to these two cases, other literature reports were no co-existence of skin or nails infections, or the history is unclear. To the best of our knowledge, this is the first complete case report on simultaneous dermatophytosis and keratomycosis.

**Table 1:** Literature review of studies on fungal keratitis caused by *Trichophyton* spp.

Year	Place	Species (Numbers of patients)	Cause	Identification method	Co-existence infections	Reference
1973	India, Jaipur	*Trichophyton spp.* (1)	Trauma	–	No	[28]
2001	Anhui, China	*T. mentagrophytes* (1)	–	Culture	Eyelids infection	[20]
2003	Oman	*T. mentagrophytes* (1)	Trauma	Culture + urease test	No	[22]
2005	Zhejiang, China	*T. mentagrophytes* (2); *T. verrucosum* (1)	–	Culture	Unclear	[27]
2006	Riyadh, Saudi Arabia	*T. schoenleinii* (5)	–	Smear + culture + histopathology	No	[29]
2010	Croatia	*Trichophyton* spp. (1)	Contact lens use	Culture + histopathology	No	[30]
2011	Saudi Arabia	*Trichophyton* spp. (1)	Trauma	Smear + culture + histopathology	No	[31]
2012	Saudi Arabia	*T. schoenleinii* (12); *T. verrucosum* (2); *Trichophyton* spp. (14)	–	Direct smear + culture	Unclear	[21]
2014	Delhi, India	*T. rubrum* (1); *T. erinacei* (1)	Trauma	Culture + biochemical identification	No	[26]
2014	Korea	*Trichophyton* spp. (1)	–	Smear + culture	Onychomycosis	[32]
2018	Changchun, China	*T. interdigitale* (1)	–	Smear + culture + ITS sequencing	Dermatophytosis	Present study

In recent years, with the development of molecular biological technologies, it has been found that species such as *T. rubrum* and *T. violaceum*, which are clinically different and very easy to distinguish in culture, are nevertheless molecularly very similar. *T. soudanense* and *T. yaoundei* are difficult to distinguish from *T. violaceum* with standard barcoding genes. Moreover, species that have long been regarded as a single species, have now been identified as a complex consisting of several molecularly similar species, such as the *T. rubrum* complex and *T. mentagrophytes* complex [9]. Depending on the type of host, the *T. mentagrophytes* complex includes the anthropophilic species *T. interdigitale* and *T. tonsurans* and the zoophilic species *T. mentagrophytes* and *T. equinum* [9]. In line with the latest taxonomic changes, previous clinical isolates of *T. mentagrophytes* should be renamed *T. interdigitale*, since the zoophilic *T. mentagrophytes* rarely infects humans and mainly causes infections in rats and camels [33]. Therefore, we speculate that corneal infections that were reported in the past as having been caused by *T. mentagrophytes* were in reality caused by the same species reported in the present study.

With the gradual deepening of the research on the relationship between immunodeficiency and fungal infections, an increasing number of studies have confirmed that genetic mutations in the innate immune system may lead to invasive fungal infections. Recent studies have shown that inherited *CARD9* [2, 3, 13] and *STAT3* [14] mutations predispose to deep dermatophytosis. In our patient, no disease-related mutation was found in the exons of *CARD9* or *STAT3*. Furthermore, none of the patient’s family members showed any symptoms of fungal infection. However, the patient’s T-cell function was abnormal, the existence of acquired immunodeficiency remains to be verified in this patient. The patient has a long history dating back to 4 years before the current visit. Although it was diagnosed as fungal keratitis in a local hospital 2 years ago, the condition was worsened because the patient did not follow the doctor’s advice. In addition, the patient has been misdiagnosed as psoriasis, eczema, and glucocorticoids have been used locally and systematically. Therefore, we speculate that poor economic and sanitary conditions, insufficient attention to the disease, and long-term misdiagnosis and corticosteroid used history were mainly responsible for the prolonged course, extensive lesions and poor prognosis. We repeatedly attempted to elicit a history of corneal trauma from the patient, but he firmly denied it. Since the self-reported primary skin lesion was on the left eyebrow, it is likely that the corneal infection was caused by the spread of the local skin infection.

*Trichophyton* species have been reported to secrete a variety of proteases and collagenases that hydrolyze keratin, collagen, and gelatin. The cornea and conjunctiva are histopathologically homologous to the epidermis. Although the former two have no horny layer, they can express keratin, and all three structures can be infected by *Trichophyton* species. Through the secretion of an extracellular collagenase with keratinolytic potential, *Trichophyton* species can cause severe stromalysis, leading to loss of vision [32]. Any breach of the corneal epithelial cell layer facilitates the penetration of fungi into the stroma. This further damages eye integrity and results in loss of function. Invasion of the anterior chamber heralds the onset of complications, and surgery is often required to eliminate the infection. Few studies have investigated the mechanisms underlying the dermatophyte infection of the human cornea. Hence, further clinical observations and experimental research are needed.

In the present study, we have reported a case of simultaneous dermatophytosis and keratomycosis caused by *T. interdigitale*, and reviewed the literature on corneal infection caused by *Trichophyton* species. Early diagnosis and prompt treatment may help improve the management and outcomes. Potassium hydroxide examination is a rapid, simple, and essential investigation for this condition. Mycological culture not only further confirms the diagnosis but also provides credible evidence to correct assertions when the result of potassium hydroxide microscopy is negative. Early diagnosis and aggressive medical treatment are of the utmost importance to improve therapeutic outcomes.

## Data Availability

The datasets used and/or analysed during the current study are available from the corresponding author on reasonable request.

## Abbreviations

CARD9: Caspase recruitment domain-containing protein 9
ITS: Internal transcribed spacer region
PCR: Polymerase chain reaction
STAT3: Signal transducer and activator of transcription 3
